# Surface modification of highly hydrophobic polyester fabric coated with octadecylamine-functionalized graphene nanosheets

**DOI:** 10.1039/d0ra02655g

**Published:** 2020-07-01

**Authors:** Ghizlane Achagri, Younes Essamlali, Othmane Amadine, Mohamed Majdoub, Achraf Chakir, Mohamed Zahouily

**Affiliations:** Laboratoire de Matériaux, Catalyse et Valorisation des Ressources Naturelles, URAC 24, FST, University Hassan II-Casablanca Morroco; VARENA Center, MAScIR Foundation, Rabat Design Rue Mohamed El Jazouli, Medinat El Irfane 10100-Rabat Morroco m.zahouily@mascir.com

## Abstract

This study focuses on the design of highly hydrophobic polyester fabrics (PET) coated with organophilic graphene nanosheets (G-ODA) through a simple, cost-effective and scalable coating method. The organophilic graphene oxide was successfully synthesized by covalently grafting a long chain fatty amine on its surface and was fully characterized by various physicochemical techniques. G-ODA was coated at different loadings onto the PET fabric ranging from 1 to 7 wt% to produce uniformly dispersed PET@G-ODA fabrics with multifunctional performances. FTIR has confirmed the formation of strong interfacial interaction between the PET and G-ODA functional groups. Moreover, the produced PET@G-ODA fabrics resulted in achieving enhanced thermal stability as well as excellent water repellency compared to the pristine PET. Water contact angle measurements showed a tremendous enhancement of surface hydrophobicity up to 148° with 7 wt% loading of G-ODA. Tensile strength tests revealed that our fabric exhibited excellent mechanical properties compared to neat PET. In addition, the designed PET@G-ODA fabrics demonstrated excellent oil/water separation efficiency for different oil/water mixtures. The obtained results are very promising in terms of designing and producing functional PET fabrics with improved thermal and surface proprieties.

## Abstract 2

This study focuses on the design of highly hydrophobic polyester fabrics (PET) coated with organophilic graphene nanosheets (G-ODA) through a simple, cost-effective and scalable coating method.

## Graphical abstract

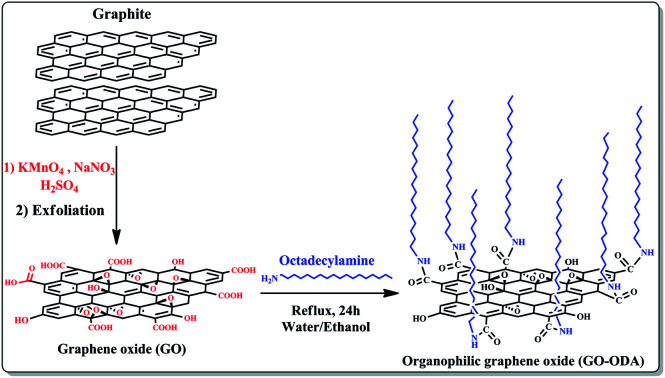

## 1. Introduction

Textile materials have been extensively used thanks to their outstanding properties, such as low cost, good flexibility, light weight, good feel, easy processing features and high porosity which is required in various applications.^1–4^ However, their utilization for various industrial and domestic applications is restricted because of their low thermal stability and hydrophilic surface; they can be quickly wetted by various liquids including water and this subsequently limits their usage for several applications. Therefore, modification of the textile fabric is of interest to improve the quality of the textile material and expand its use for multiple applications. Recently a large number of researches have reported coated textile fabrics with different materials to introduce several significant multifunctional properties such as: electrical conductivity, ultraviolet blocking, photo catalytic properties, photo-thermal conversion,^5^ far-infrared emission^6–8^ and hydrophobicity.^9–13^

Hydrophobic textiles akin to lotus leaf and rose petal surfaces have recently attracted so much attention,^14–17^ and offer a significant potential for implication for various scientific and industrial applications. Several methods of coating have been developed to mime the high hydrophobicity on the textile fabrics, among them: solution-immersion coating, sol gel based coating, wet-chemical process, spray coating^18–24^*etc.* However, most of these methods have limitations in large scale applications due to their high cost, toilsome fabrication processes and could be employed effectively depending on a requirement for particular applications. In this context, the major challenge is to elaborate a simple and scale-up approach to produce durably high hydrophobic textile fabrics without damaging their native properties. Dip coating method^25–28^ is recognized to be a promising alternative due to its facility, reproducibility and suitability for mass production in a large scale.

Recently, graphene and related materials have attracted enormous attention in the scientific community during the past decades in view of their unique, unprecedented and countless physicochemical properties that have permitted access to these materials in almost every domain,^29–32^ including textile coating.^33,34^ One of the most effective and widely used approaches to synthesis graphene could be chemical reduction of graphene oxide (GO). Furthermore, the presence of various kinds of oxygen-based functional groups on the GO basal planes and edges (hydroxyl, carboxyl, oxygen functional groups and epoxy) provide active sites for chemical functionalization to achieve new fascinating proprieties such as hydrophobicity, oleophobicity, superhydrophobicity and so on. For instance, Lin *et al.* synthesized functionalized graphene oxide by covalently grafting octadecylamine on its surface.^35^ They reported that the octadecylamine functionalized GO achieved high hydrophobicity with a water contact angle up to 132.4°. Moreover, Xu *et al.* reported the synthesis of hydrophobic modified graphene oxide with a water contact angle of 125.8°.^36^ The overall approach is to introduce new chemical moieties to hydrophobize GO's surface which implies a decrease in the surface energy thus highly hydrophobic GO based materials can be obtained.^37^ Yet, the critical challenge of GO functionalization is to carry the procedure so that it only modifies its surface, while preserving its intrinsic proprieties *i.e.* structural and morphological characteristics. So far, few studies have been conducted on the functionalization of graphene oxide to enhance the hydrophobicity of polyester based textile materials.^33,38^

In the other hand, the international maritime traffic of oils continues to increase in order to meet industrial and food demands. Moreover, the increase in the world's population, which consumes oil for its food and the diversification of industrial applications involving the processing of oils, especially in cosmetics and pharmaceuticals industry, leads to intense traffic inevitably oil spill accidents.^39,40^ Despite the taken measures by the International Maritime Organization (IMO) to make oils transportation safer and protect the environment by regulating discharges. Accidents resulted in the dumping of oils such as rapeseed oil into the Port of Vancouver in 1989 and in 1998 in Hong Kong city. Wales in 1991 (Mudge, 1995), palm kernel oil in 1997 in the channel or palm oil in the Mississippi River (USA) in 1998. Oils, although inherently non-hazardous, have environmental and ecological effects on the marine fauna and flora, because of the importance of the spilled quantities. The spread-out layers of oil, can drift over several tens of nautical miles, emulsify to form a product that is difficult to remove. However, prevention of damage to the environment can be facilitated through a development of new energy-efficient solutions, which can enhance and control specific wettability and absorption. Hydrophobic surfaces have drawn a lot of interest lately as for the oil/water separation due to their capacity to repelling water and absorbed only oil.

In this respect, the fabrication of highly hydrophobic polyester fabrics was achieved using the dip coating approach in an organophilic graphene oxide solution followed by an *in situ* chemical reduction. The organophilic graphene oxide (GO-ODA) was synthesized *via* chemical grafting of octadecylamine and was fully characterized by various physicochemical techniques. Optical, structural, thermal, morphological, and mechanical characterizations of the produced PET@G-ODA fabrics were performed by means of FTIR, TGA, SEM and tensile strength. Furthermore, water contact angle measurement was also performed to characterize the water-repelling property of treated fabrics. Since the produced PET@G-ODA fabrics have the potential to be used in numerous domains such oil/water separation, their separation performances for different oil/water and organic solvents mixtures was also investigated and discussed. The obtained findings showed the effectiveness of GO-ODA coating achieving enhanced proprieties compared to the pristine PET while maintaining the intrinsic characteristics of the fabric such as lightweight and good feel.

## 2. Experimental

### 2.1. Materials and apparatus

Knit polyester fabric (100 g m^−2^) was processed for coating. Graphite powder (particle size > 45 μm) with purity upper to 99.99% was purchased from Sigma-Aldrich. All the other chemical agents including, sulfuric acid (H_2_SO_4_), potassium permanganate (KMnO_4_), sodium nitrate (NaNO_3_), hydrogen peroxide (H_2_O_2_), octadecylamine (C_18_H_39_N), tetrahydrofurane (C_4_H_8_O) and sodium hydrosulfite (Na_2_S_2_O_4_) were supplied by Sigma-Aldrich.

### 2.2. Preparation of graphene oxide nanosheets

**Fig. 1:**
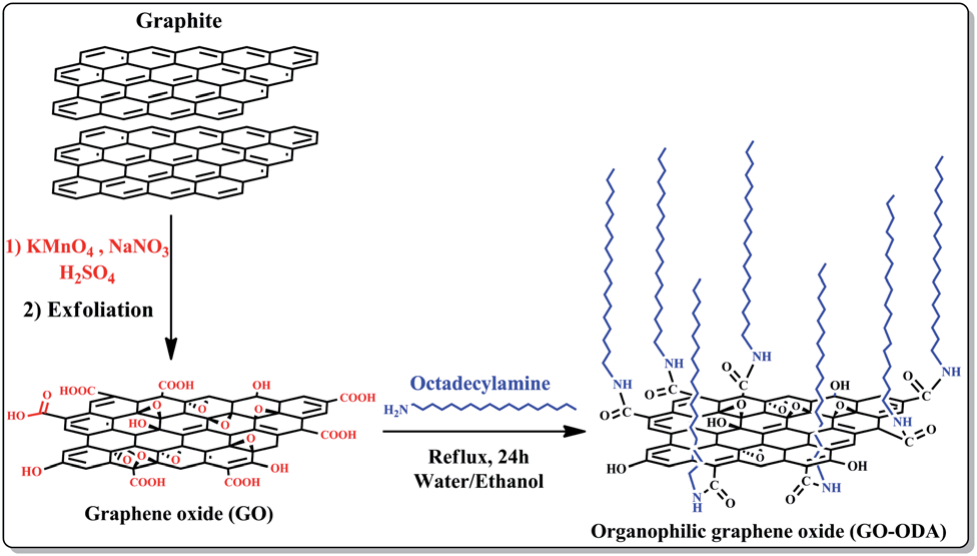
Schematic illustration of the processes involved in fabrication of the chemically modified graphene oxide.

In the present work, the graphene oxide was synthesized from natural graphite following modified Hummer's method.^41^ The obtained aqueous graphene oxide dispersion was used for the preparation of ogranophilic graphene oxide nanosheets (GO-ODA). The preparation process of GO-ODA is illustrated schematically in Fig. 1. In short, 200 mg of graphene oxide nanosheets (GO) was dispersed in 20 mL of distilled water. In the mean time 200 mg of octadecylamine (ODA:CH_3_(CH_3_)_17_–NH_2_) was dissolved in 20 mL of ethanol. Then, ODA suspension was added to the GO solution and the mixture was refluxed for 24 h, during which GO nanosheets react with ODA molecules to form GO-ODA. Upon reaction completion, the solid product was repeatedly washed with ethanol to remove unreacted ODA moieties then isolated by centrifugation and finally dried in a vacuum oven at 70 °C.^42^

### 2.3. Preparation of G-ODA coated knit polyester fabric (PET@G-ODA)

**Fig. 2:**
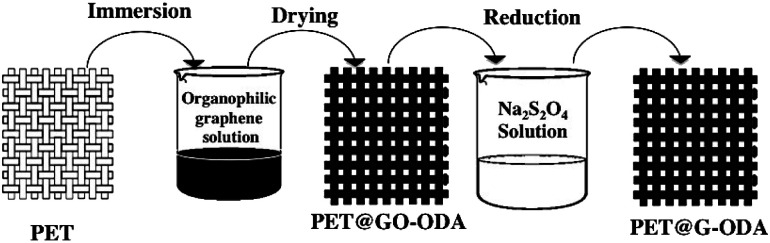
Preparation process of PET@G-ODA.

The PET@G-ODA samples have been prepared using an easy “dip-coating” approach followed by *in situ* chemical reduction. The process occurs in three consecutive steps as depicted in Fig. 2. Before coating, knit polyester with dimensions of 10 cm in length and 4 cm in width was pretreated with an aqueous solution containing 3 g L^−1^ of ammonia and 2 g L^−1^ of nonionic detergent for 1 h at 100 °C. Then, the PET fabrics were washed with distilled water and dried in an oven at 80 °C for 4 h. After that, the pretreated fabrics were dipped in an aqueous solution of exfoliated GO-ODA in tetrahydrofurane (THF) (1 mg mL^−1^) and then dried in an oven at 90 °C for 2 h. This process of dip coating and drying was performed several times in order to increase the amount of GO-ODA loaded on the samples. In this step GO-ODA sheets were tightly deposited on the surface of the PET fabrics. An obvious color change from white to black was visualized as the number of coating cycle's increases. Subsequently, the as-obtained PET@GO-ODA samples were chemically reduced by simple immersion in 100 mL aqueous solution of Na_2_S_2_O_4_ (50 × 10^−3^ M) while stirring for 1 h at 90 °C to reduce GO-ODA into G-ODA.

### 2.4. Characterization techniques

The chemical structure of the GO-ODA and the produced PET@G-ODA fabrics was studied by infrared spectroscopy using an Affinity-1S SHIMADZU spectrometer equipped with a golden gate single reflection attenuated total reflectance (ATR) accessory. FTIR spectra were recorded in the range of 500–4000 cm^−1^ at a resolution of 16 cm^−1^. X-ray diffraction (XRD) measurements were performed on a Bruker D8 Discover diffractometer fitted with a Cu-Kα irradiation source (*λ* = 1.5438 Å) operating at 40 kV with a scan speed of 2° min^−1^. The color changes of the produced fabrics were measured by color coordinates (L*, a*, b*) and reflectance spectra using color spectrophotometer BYK in the spectral range 400–700 nm with 20 nm resolution. The surface morphology of PET@G-ODA was observed by Scanning Electron Microscopy (SEM), using an FEI Quanta 200-ESEM operating at an accelerating voltage of 20 kV. The thermal stability of the uncoated and coated polyester fabric was investigated by thermo-gravimetric analysis (TGA) using a TGA-Q500 (TA instruments) at the heating rate of 10 °C min^−1^ in the air atmosphere from 25 to 700 °C. The water contact angles measurements of the polyester fabrics were determined using an automatic video contact-angle testing apparatus (OCA 40, DATAPHYSICS) by the sessile drop method. A drop of 3 μL of distilled water was applied on the surface of the PET@G-ODA and the contact angle was determined from the video camera images of the drop. The tensile properties of the PET@G-ODA fabrics were determined on a Shimadzu EZ-SX apparatus, at a tensile speed of 10 mm min^−1^. Measurements were performed upon rectangular specimens of 80 mm length and 40 mm width and 0.2 mm thickness.

## 3. Results and discussion

### 3.1. Characterization of GO-ODA

The crystal structure of all the samples was determined using X-ray diffraction (XRD). Fig. 4 shows the XRD patterns of natural graphite, graphite oxide (GO) and organophilic graphene oxide (GO-ODA). XRD diffractogram of graphite powder exhibits a strong and sharp diffraction signal localized at 2*θ* = 26.5° assigned to the (002) reflection of an arranged graphite structure (JCPDS no. 75-1621),^43^ and corresponding to an interlayer distance (*d*_002_) of 0.33 nm calculated by Braggs law. After oxidation and ODA functionalization, the diffraction peak observed at 2*θ* = 26.5° has shifted to 2*θ* = 21.05°, indicating an increase in the interlayer *d*-spacing (0.42 nm) compared to natural graphite (0.33 nm), this increase in the interlayer spacing is attributed to the covalently attachment of long chain fatty amine ODA on the GO surface moieties^44^ through an amidation reaction. The main reactive sites for the chemical grafting of ODA molecule onto GO are the epoxy groups and the carboxyl groups located at the basal and the edges of the graphitic structure.^45^ For G-ODA the *d*-spacing shifts to 0.44 nm with 2*θ* = 20.16°, suggesting that during the chemical reduction, ODA molecules were bonded to the exogenous groups of reduced graphene oxide and prevented the nanosheets from agglomeration.

**Fig. 3:**
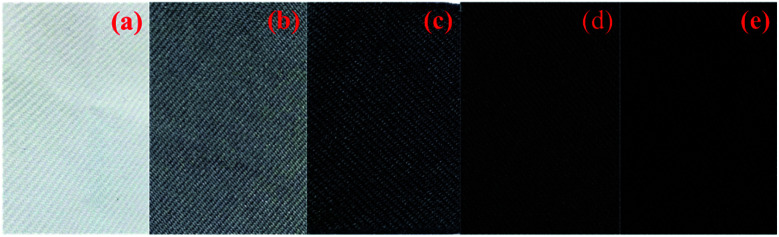
Photo images for the PET samples: (a) neat PET, (b) PET@G-ODA-1%, (c) PET@G-ODA-3%, (d) PET@G-ODA-5% and (e) PET@G-ODA-7%.

**Fig. 4:**
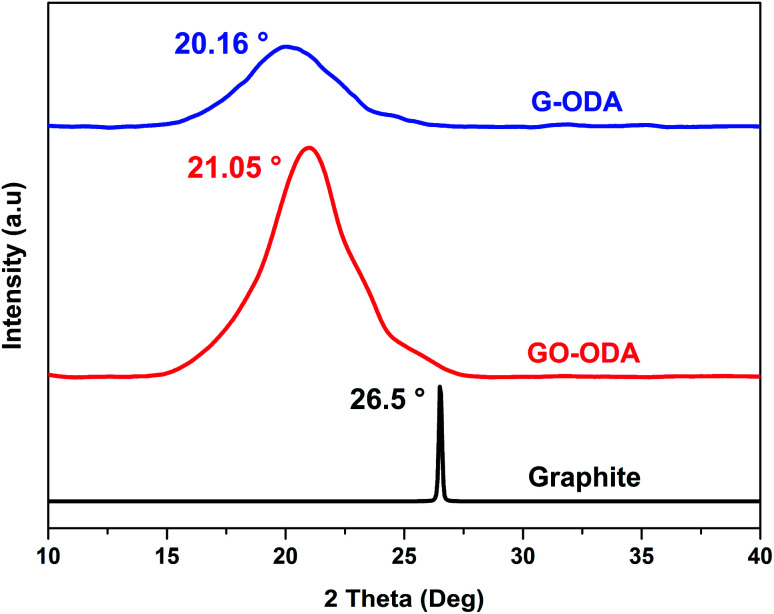
XRD patterns of graphite, GO-ODA and G-ODA.

**Fig. 5:**
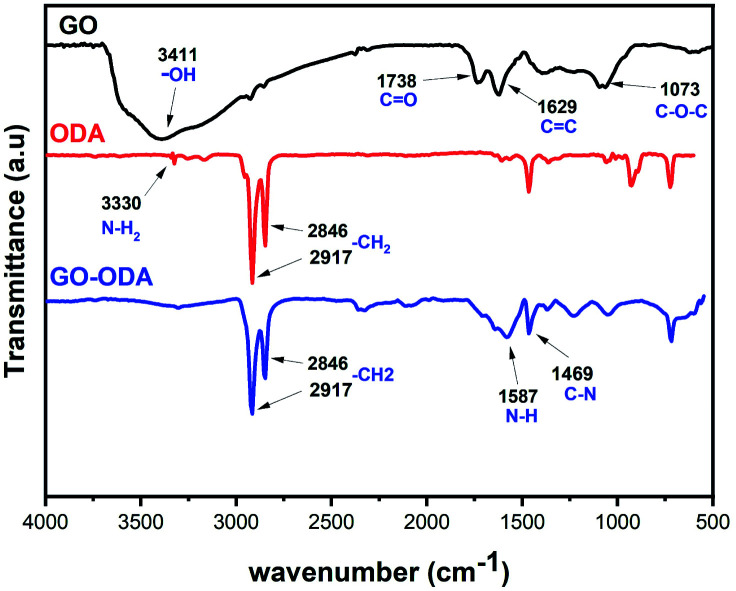
FT-IR spectra of GO, ODA and GO-ODA.

FTIR analysis was performed to define the chemical changes that can possibly happen during the chemical reaction between GO and ODA. Fig. 5 showed FTIR spectra of GO, ODA and GO-ODA. As expected, the GO spectrum shows the specific absorption bands of GO that appear respectively at 3411, 1738, 1629, and 1073 cm^−1^, corresponding to the hydroxyl groups, C=O in carboxyl group, C=C in aromatic ring, and C–O–C in epoxide.^46^ The existence of these functional groups endows GO with possibilities for functionalization. After the chemical reaction with ODA, the successful grafting of ODA molecule on GO is clearly observed *via* the emerging bands at 2917 cm^−1^ and 2846 cm^−1^ (–CH_2_ stretching in the octadecyl chain).^47^ The new bands at 1587 and 1469 cm^−1^ arising, respectively, from N–H bending and C–N stretching of amide in GO–ODA gave further evidence to the successful amidation reaction between carboxyl groups of GO and the amine groups (NH_2_) of ODA molecules. Moreover, the decrease in the relative intensity of the peaks observed at 1073 and 1738 cm^−1^ (C–O–C/–COOH) suggests that these groups are involved in a chemical reaction with the amine group of ODA molecule through an amidation reaction. We believe that the reaction between GO and ODA occur favorably between the COOH groups of GO and NH_2_ groups of ODA, but it could also occur *via* nucleophilic substitution between epoxy group of GO (C–O–C) and the amine group of ODA (NH_2_). In this case the NH_2_ group of ODA molecule serves as a nucleophile and attacks the carbon atom in epoxy group leading to oxirane ring opening reaction, thus grafting the ODA hydrocarbon chains in GO surface. These results confirmed the chemical grafting of ODA into GO's surface, which is similar to what it was reported in previous works. These results confirmed the chemical grafting of ODA into GO's surface, which is similar to what it was reported in previous works.^48,49^

### 3.2. Characterization of PET@G-ODA

Fig. 3 showed the digital images of PET, PET@G-ODA-1%, PET@G-ODA-3% PET@G-ODA-5% and PET@G-ODA-7%. It is clear from the photographic images that the color of PET fabric was significantly affected after coating with G-ODA. The blank sample, PET turned from white to dark grey after dipping in G-ODA solution, and changed to black with increased dipping cycles to reach 7% of G-ODA loaded. These observations indicate that the coating of PET fabrics with G-ODA was successfully occurred.

The color changes of the PET fabrics were measured by color coordinates (L*, a*, b*). Table 1 illustrates color coordinates (data COLOR) of coated and uncoated polyester samples. It was found that color coordinates values changed after G-ODA coating, indicating the color differences between the pristine PET samples and the coated ones. The pristine PET shows the highest lightness (L* = 88.74) with values of a* (redness-greenness) and b* (yellowness-blueness) equal to 0.91 and 2.41, respectively, describing its white color. After coating PET samples with G-ODA, an obvious decrease was observed in L* values (31.56, 29.33, 19.02 and 18.90) respectively, for the PET@G-ODA-1%, PET@G-ODA-3%, PET@G-ODA-5% and PET@G-ODA-7%, and for a* values (from 0.91 for the pristine PET to 0.23 for the PET@G-ODA-7%). Moreover, by comparing the L*a*b* values for each sample, it can be noticed that the samples don't match in color and that the greater the amount of G-ODA is, the darker is the PET@G-ODA fabric, confirming the successful coating of the PET fabric.

**Table 1:** Color coordinates (data COLOR) of the pristine PET fabric and the treated PET@G-ODA

Samples	L*	a*	b*
PET	88.74	0.91	2.41
PET@G-ODA-1%	31.56	0.46	1.85
PET@G-ODA-3%	29.33	0.63	1.59
PET@G-ODA-5%	19.02	0.35	0.52
PET@G-ODA-7%	18.90	0.23	0.7

**Fig. 6:**
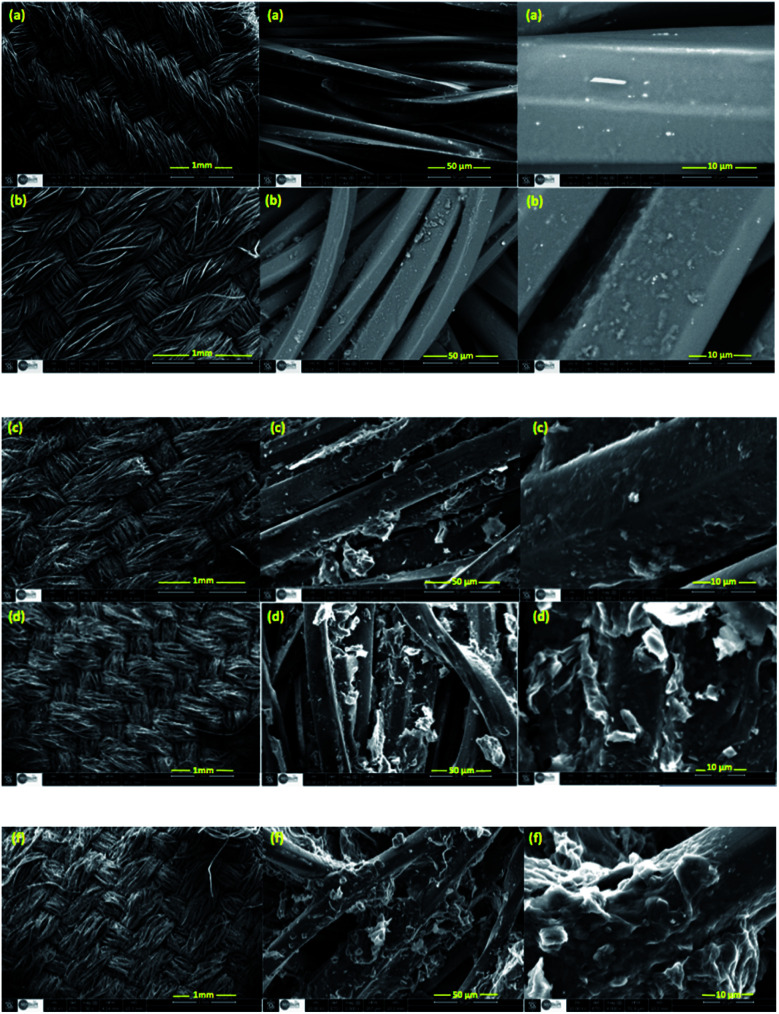
SEM images of PET (a), PET@G-ODA-1% (b), PET@G-ODA-3% (c), PET@G-ODA-5% (d) and PET@G-ODA-7% (f).

The Scanning Electron Microscope (SEM) was employed in order to investigate the fibers and the surface morphology of treated and untreated fabrics. Fig. 6 shows the SEM images of PET, PET@G-ODA-1% (b), PET@G-ODA-3%, PET@G-ODA-5% (d) and PET@G-ODA-7%. From this figure, it can be clearly seen that the surface morphology of the uncoated polyester fabric (PET) (Fig. 6a) presents a relatively smooth and sleek fibers texture. After coating, G-ODA sheets were clearly deposited onto the surface of PET fabrics and also inserted into the pores between fiber–fiber of PET fabrics, as shown in Fig. 6b–f. The surface of the fabric's fibers became completely covered with the rough layer of G-ODA on each fiber (Fig. 6b–f). These results confirm the successful attachment of G-ODA on the surface of PET. In addition, SEM images of PET@G-ODA fabrics revealed that the PET fibers were almost intact after coating with G-ODA, which is indicating that the fabrics did not undergo structural changes during the coating. From all of this results, coating PET with G-ODA can generate a roughness on the surface of PET fibers that resembles the surface texture of a lotus leaf or a rose petal, and thus it provides the responsible texture leading to the formation of high hydrophobic surfaces, based on recent investigations.^50,51^

**Fig. 7:**
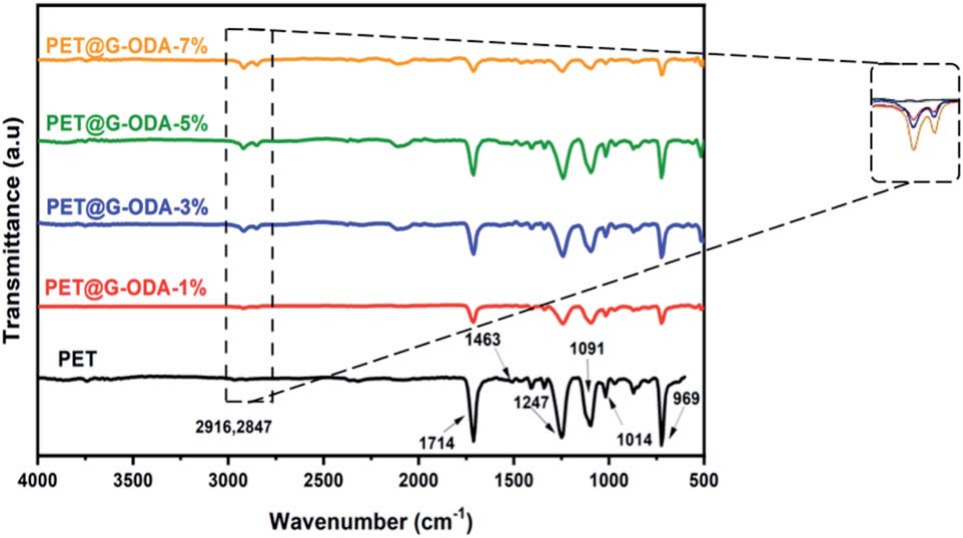
FT-IR spectra of PET, PET@G-ODA-1%, PET@G-ODA-3%, PET@G-ODA-5% and PET@G-ODA-7%.

The FTIR spectrum of the uncoated knit polyester fabric (Fig. 7), shows characteristic bands at 969 and 1014 cm^−1^ corresponding to the C–O stretching of glycol and benzene in-plane vibrations, respectively. The bands observed at 1091 and 1247 cm^−1^ represent the ester C=O stretching vibration and the band centered at 1714 cm^−1^ is assigned to C=O group of aromatic ester.^52^ The absorption bands at about 1460 cm^−1^ is attributed to C=C stretching vibration.^53^ The FT-IR spectra of PET@G-ODA samples revealed some noticeable changes including notable reduction in the intensity of carbonyl groups stretching vibration (1714 cm^−1^) suggesting that G-ODA strongly interacted with the PET fabric, occurring probably between the ester group of PET and NH, C–N group of the amide, or through hydrophobic interaction between G-ODA molecules and C=C and benzene rings of PET that interact through π–π stacking,^54^ leading to the formation of a uniform G-ODA coating on PET surface. Furthermore, the apparition of a new absorption bands at 2847 and 2916 cm^−1^ assigned to the symmetric and asymmetric stretching vibration modes of CH_2_ groups in octadecylamine grafted onto GO's surface gave further proof to the establishment of strong interactions between the PET and G-ODA.

**Fig. 8:**
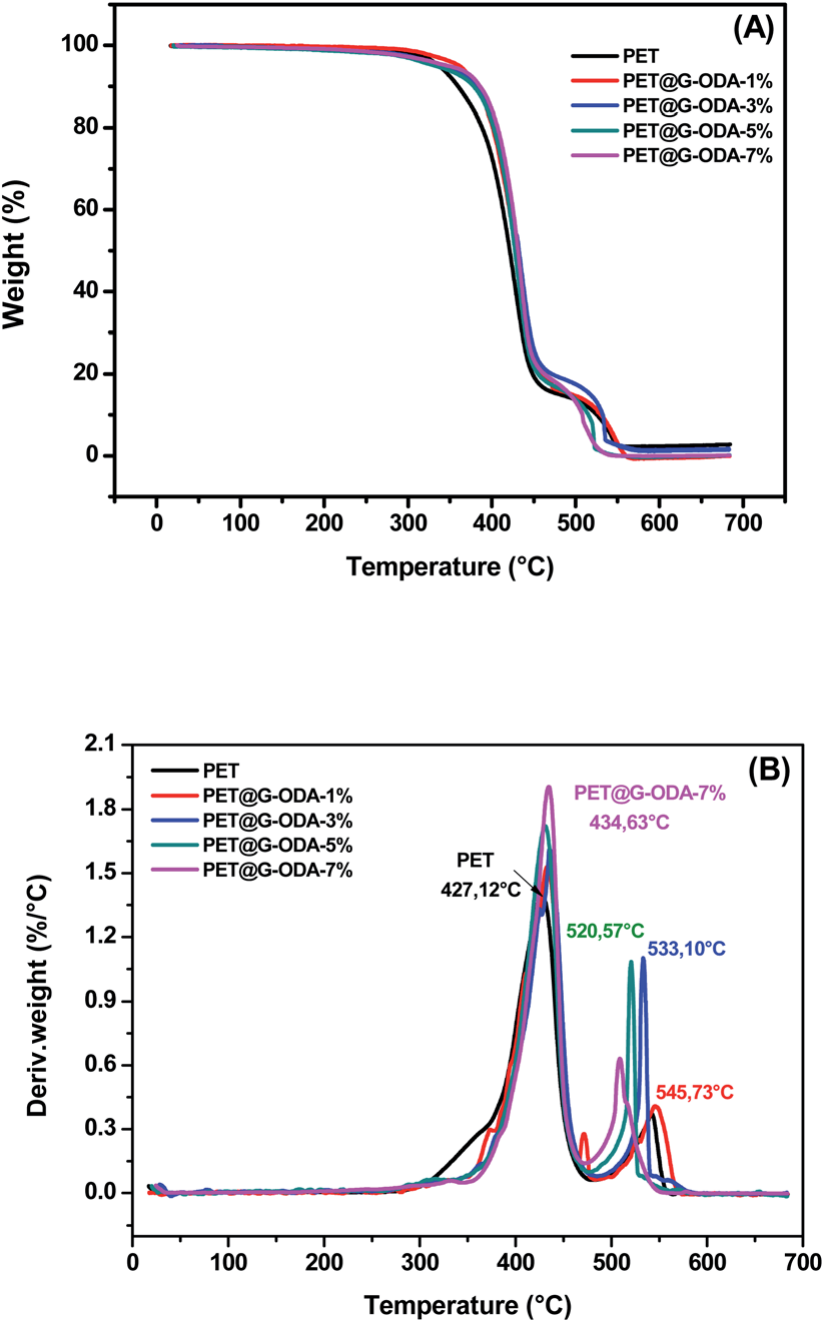
(A) TGA and (B) DTG curves of PET, PET@G-ODA-1%, PET@G-ODA-3%, PET@G-ODA-5% and PET@G-ODA-7%.

The thermal degradation behavior of PET and PET@G-ODA fabrics was investigated by means of TGA and DTG with 10 °C min^−1^ heating rate under air atmosphere. TGA curves of all the samples being presented in Fig. 8, while the different parameters obtained from the thermograms are listed in Table 2. It is clearly observed that all the samples, regardless the G-ODA content, undergo mainly two distinct decomposition stages and were identified as follows, (i) a primary degradation step was occurred in the range of 300–470 °C corresponding to the ester linkage decomposition in the PET fabric which took place *via* the depolymerization of polyester through chain scission producing carboxy- and vinyl-terminated chain fragments^55^ simultaneously occurring with the decomposition of both physically adsorbed ODA molecules remaining after washing by ethanol and chemically grafted ODA chains, (ii) the second degradation step undergoes up to 600 °C corresponding to C–C bond cleavage leading to the formation of volatile compounds such as carbon mono- and dioxide, methane, ethylene, benzene, benzaldehyde, formaldehyde and acetaldehyde, as well as the pyrolysis of the residual covalently grafted ODA chains. Compared to pristine PET, the PET@G-ODA fabrics displayed enhanced thermal stabilities, which can be ascribed to the strong interactions established between the G-ODA coating and the PET fibers. Overall, the thermal decomposition of PET@G-ODA fabrics took place at higher temperature than that of uncoated PET. The significant increase in *T*_10%_ (358.6 °C for PET *vs.* 386.7 °C for PET@G-ODA 7%) and *T*_80%_ (448.9 °C for PET *vs.* 463.2 °C for PET@G-ODA 7%) gave further supports that G-ODA coating has successfully improved the overall thermal stability of the PET fabric. Such enhancement can be attributed to the presence of G-ODA as coating, improving the overall thermal resistance through the establishment of strong interactions with the PET fibers. Furthermore, this improvement may be due to the contribution of graphene's intrinsic properties that improves the thermal properties of textile fabrics.^56^

**Table 2:** *T*_10%_ and *T*_80%_ of PET, PET@G-ODA-1%, PET@G-ODA-3%, PET@G-ODA-5% and PET@G-ODA-7%

	PET	PET@G-ODA-1%	PET@G-ODA-3%	PET@G-ODA-5%	PET@G-ODA-7%
*T*_10%_ (°C)	358.69	382.45	380.04	377.63	386.75
*T*_80%_ (°C)	448.90	458.37	472.83	454.23	463.21

**Fig. 9:**
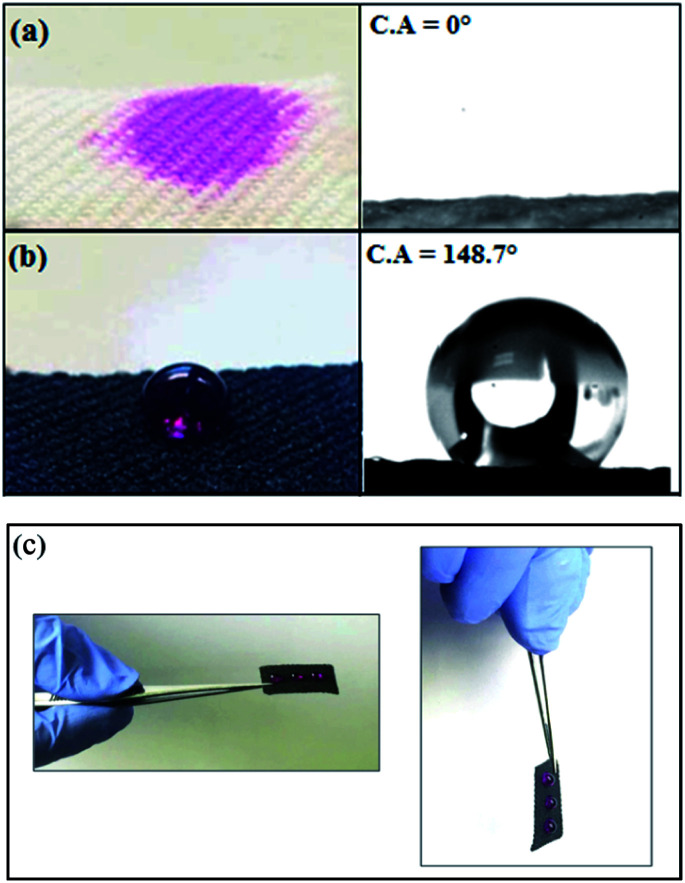
Demonstration the hydrophobic character of the PET@G-ODA-7% fabric compared to pristine PET: (a) pristine PET, (b and c) PET@G-ODA-7%.

**Fig. 10:**
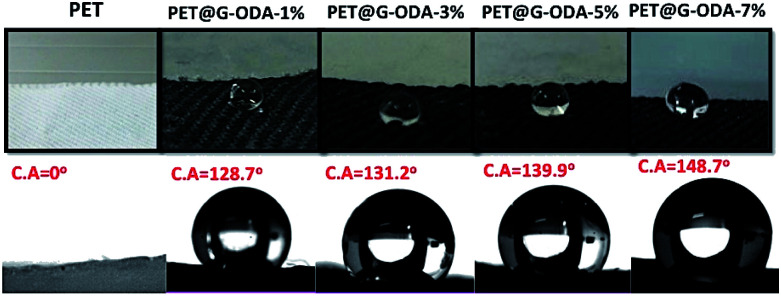
Water contact angle values of PET, PET@G-ODA-1%, PET@G-ODA-3%, PET@G-ODA-5% and PET@G-ODA-7% at *T* = 0 s.

As reported previously, the surface roughness plays an important role in determination of the material's wetting properties. According to Wenzel model,^57^ the surface roughness of a material will usually increase its apparent hydrophobicity. The influence of surface roughness on the high hydrophobicity and wetting property of PET@G-ODA fabrics is affirmed by the contact angle measurements (Fig. 9). As shown in Fig. 9a, the native PET textile was rapidly wetted by water droplets with apparent contact angle of 0°, this “hydrophilicity” is due to the fact that water droplet tend to diffuse through the woven PET fabric *via* the wicking effect^58^ and capillary force governed by the porous structure of PET fabric. After coating with G-ODA (Fig. 9b), all the PET samples turned to be highly hydrophobic which can be explained by the deposition of graphene moieties bearing long hydrophobic hydrocarbon chains on the PET surface.^37,59^ Water droplets with spherical shape appeared to remain durable on the treated textile's surface, this property was achieved for all loaded samples and the contact angle value increases from 128.7 to 148.7° by increasing G-ODA contents from 1 to 7% (Fig. 10). Moreover, a drop on the surface of treated PET doesn't roll off even on flipping it upside down (Fig. 9c). As results, PET@G-ODA exhibits a “petal effect” which is similar to a rose petal *i.e.* hydrophobic character along with high adhesion forces (Fig. 9c).

**Fig. 11:**
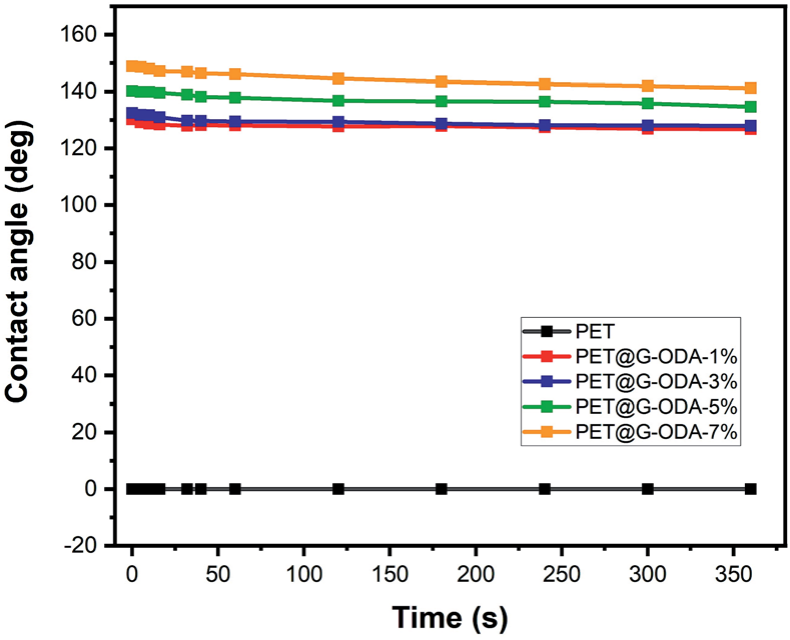
Evolution of water contact angle with time for the PET, PET@G-ODA-1%, PET@G-ODA-3%, PET@G-ODA-5% and PET@G-ODA-7% at *T* = 0 s.

The water contact angle measurement, as a function of time was monitored in order to investigate the change in the hydrophobicity of the different prepared PET@G-ODA fabrics (Fig. 11). As clearly observed in the figure, the treated PET maintained its high hydrophobicity and only a slight decrease in the contact angle for all samples was detected, suggesting the excellent durability of the PET@G-ODA textiles as highly hydrophobic surfaces.

The prepared fabrics in our study exhibit high hydrophobic properties, which make them suitable for different applications. Due to their excellent fluid absorption capacity, textile materials have been extensively used for cleaning and mopping. Therefore, to demonstrate absorption capacity of the elaborated PET@G-ODA; an oil/water separation experiment was performed. Fig. 13a shows the selective absorption of the oil from an oil/water mixture by the treated PET@G-ODA-7% sample. The baker contains frying oil (blue) and an aqueous solution of distilled water. According to this figure, it can be clearly observed that the highly hydrophobic PET@G-ODA coated samples, when immersed in the oil/water mixture, absorbed the oil selectively and completely, within a few seconds, without the requirement of extra force, leaving behind a clear aqueous solution. After the procedure, the water volume remained the same with no change, leading to a selective oil/water separation. The coated samples were found to be efficient as an absorbent and absorbed oil almost 4 times of their initial weight. The oil absorption capacity (*C*_oil_) by different samples was calculated and quantified using following formula:^60,61^*C*_oil_ (g/g) = (*W*_f_ − *W*_i_)/*W*_i_where *W*_i_: weight of the sample and *W*_f_: weight of the sample with absorbed oil.

The absorption capacities of the PET@G-ODA for other types of oil compounds and organic solvents are illustrated in Fig. 13b. The PET coated samples were found to be highly efficient as absorbents and absorbed oil more than 4 times their weight.

**Fig. 12:**
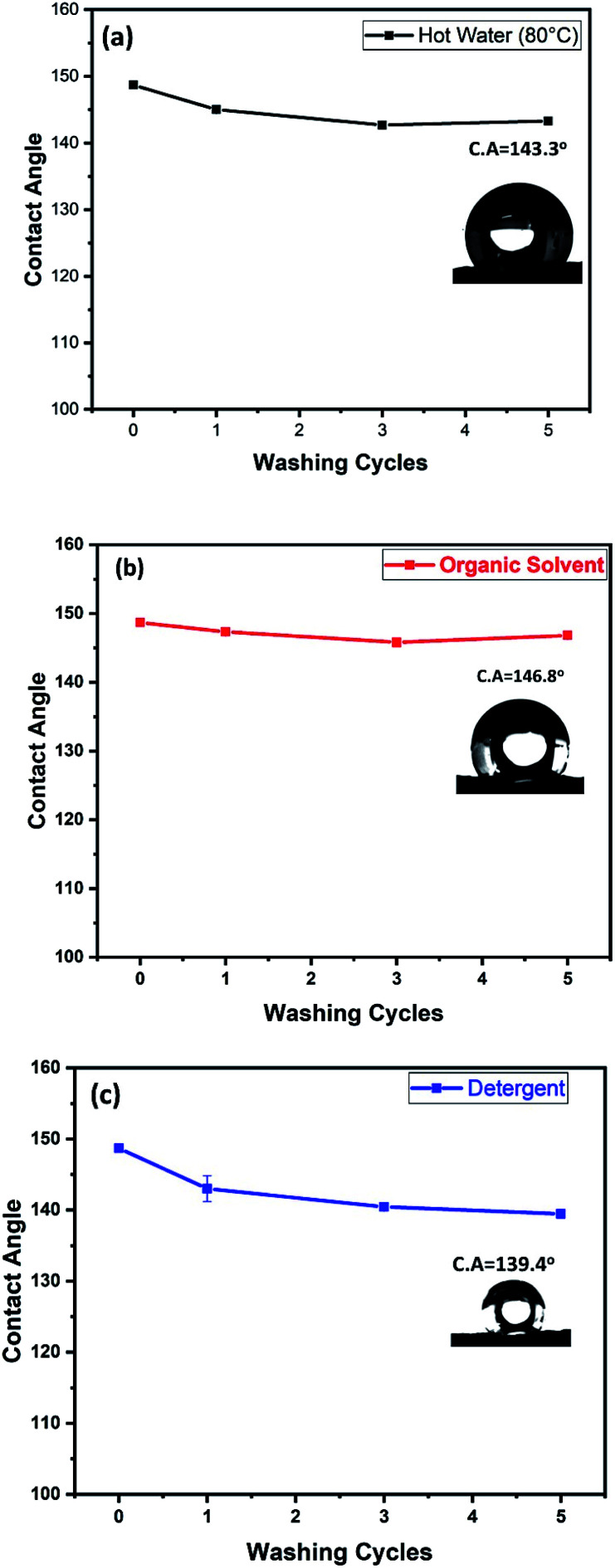
PET@G-ODA-7% washing test with (a) hot water, (b) organic solvent, (c) detergent.

Washing test was also preformed in order to investigate the PET@G-ODA coated samples stability after washing cycles. As known, the ultrasound treatment would rapidly deteriorate the coating structure and speed up the coating removal from the substrate. The samples were washed for 40 min each, under ultrasound treatment. The PET@G-ODA-7% was immersed in a 100 mL solution of (i) hot water, (ii) detergent and (iii) organic solvent, for 5 cycles. After each washing cycle, the sample was washed with deionized water and dried. Contact angle measurements were carried out after every washing cycle and the results are summarized in Fig. 12. PET@G-ODA-7% (C. A = 148.7°) maintained its highly hydrophobic property after washing under ultrasound treatment, with contact values of 143.3°, 146.8° and 139.4°, respectively after 5 cycles washing with hot water, THF and detergent. These results confirm the durability of G-ODA coating as well as the strong interaction between the coating and the fibers, as it was mentioned previously.

**Fig. 13:**
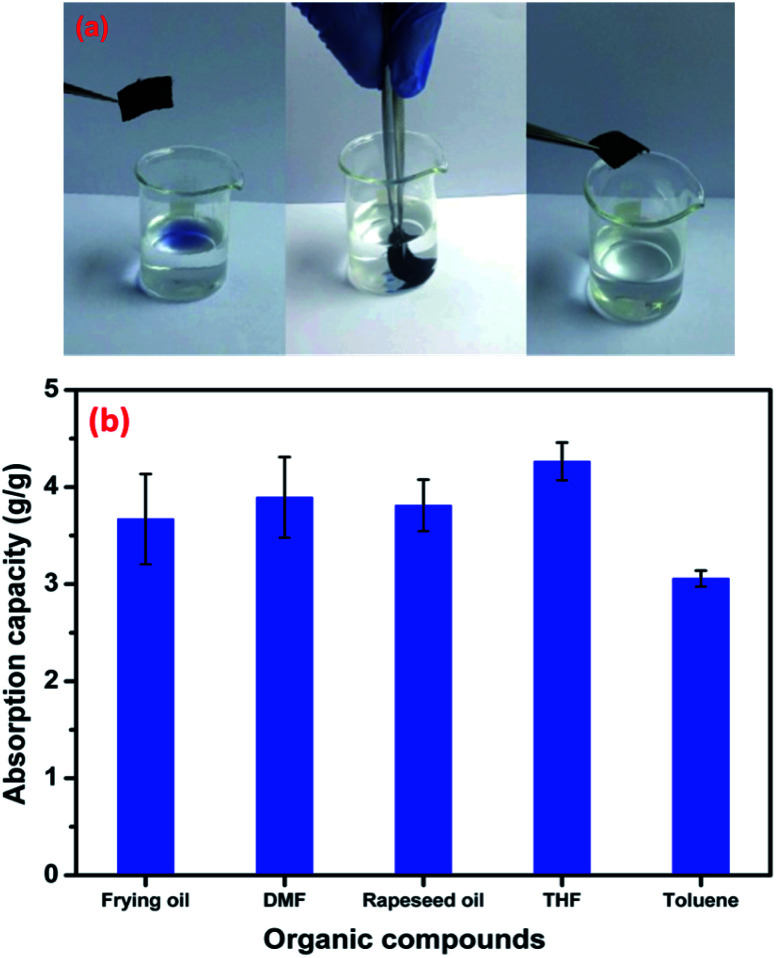
Removal of oil (blue color) from water using PET@G-ODA (a) absorption capacity of the PET@G-ODA coated sample for different organic solvents and oils (b).

It is well reported that the density and viscosity of the absorbed oil usually affect the absorption capacity of absorbents.^62,63^ They exhibit lower absorption capacity for organic compounds with high viscosity, this last resist the diffusion of oil towards the absorbent, which leads to slow absorption for oils. In the present study, the absorption capacity of the PET@G-ODA-7% coated sample was found to be higher for THF due to its low viscosity (*η* (THF) ≈ 0.46 × 10^−3^ Pa s) compared to the other used organic compounds (*η* (DMF) ≈ 0.80 × 10^−3^ Pa s and *η* (toluene) ≈ 0.59 × 10^−3^ Pa s). In the case of oils, the absorbed volumes are almost similar; this is probably due to the approaching density values of rapeseed oil and frying oil, which are 0.916 g cm^−3^ and 0.910–0.920 g cm^−3^, respectively.

The absorbed oil can be taken out of the PET@G-ODA textile easily by simple squeezing, and washing with acetone. The oil-soaked textile can be reused in order to absorb oil once more.

As mentioned previously, the dip-coating approach guaranties high homogeneity and adhesion of G-ODA to the PET samples. The aim of PET coating is adding new hydrophobic properties *via* grafting organophilic graphene functionalities into the fabric's surface, while maintaining the high flexibility, lightweight and without damaging the structure of textile fibers.

**Fig. 14:**
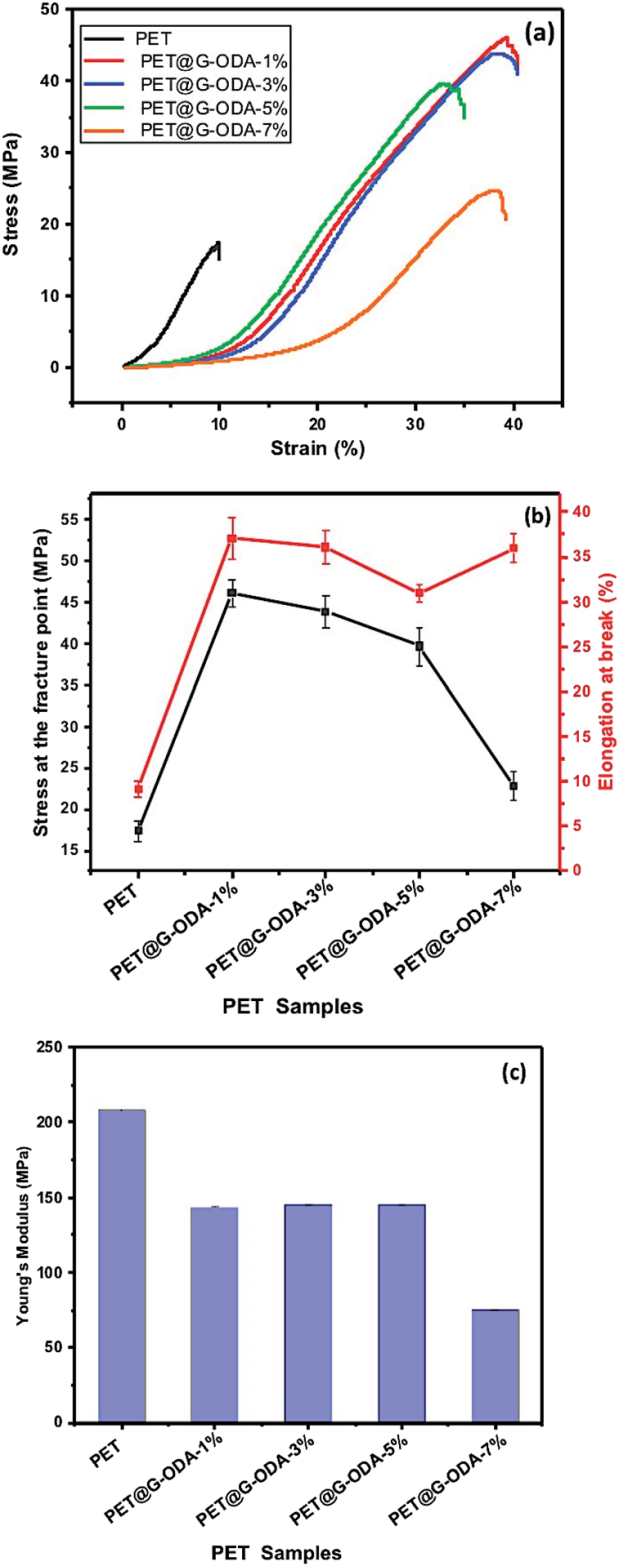
(a)Stress–strain curves, (b) elongation at break (%) and stress and the fracture point (MPa), (c) Young's modulus of pristine PET, PET@G-ODA-1%, PET@G-ODA-3%, PET@G-ODA-5% and PET@G-ODA-7%.

Tensile test was performed to investigate the mechanical behavior of the PET coated samples. The tensile properties of pristine and coated PET samples were obtained from their stress–strain curves. The average tensile strength (MPa), elongation at break (%), and Young's modulus (MPa), are calculated and illustrated in Fig. 14, and the corresponding results are represented in Table 3. According to the figures, pristine PET showed an inferior tensile strength (Fig. 14a) and elongation at break (Fig. 14b) compared to PET@G-ODA samples, indicating that G-ODA coating allows amelioration in the mechanical properties of PET fabrics.

**Table 3:** Tensile parameters of coated and uncoated PET fabrics

Samples	Stress at the fracture point (MPa)	Elongation at break (%)	Young's modulus (MPa)
PET	17.16 ± 1.19	9.16 ± 0.9	208.3 ± 0.4
PET@G-ODA-1%	46.01 ± 1.62	37.08 ± 2.01	143.6 ± 0.2
PET@G-ODA-3%	43.81 ± 1.92	36.05 ± 1.8	145.2 ± 0.2
PET@G-ODA-5%	39.62 ± 2.3	30.94 ± 1.02	145.2 ± 0.2
PET@G-ODA-7%	22.80 ± 1.7	35.90 ± 1.6	75.2 ± 0.2

Pristine PET showed a tensile strength of 17.33 ± 1.19 MPa and an elongation at break of 9.16 ± 0.9%. After only 1 wt% G-ODA loading, the tensile strength was increased by 62.33%, to obtain 46.01 ± 1.62 MPa and the elongation at break increased, as well, by 75.29% corresponding to 37.08 ± 2.01%. Remarkably, the increase of G-ODA to higher amounts (3%, 5% and 7%) on the coated PET fabrics decreases the tensile strength to 43.81 ± 1.92 MPa, 39.62 ± 2.03 MPa and 22.80 ± 1.7 MPa, respectively for PET@G-ODA-3%, PET@G-ODA-5% and PET@G-ODA-7%. But they remain higher than the uncoated PET tensile strength. In addition, the coated PET samples displayed higher elongation at break values (Fig. 14b), that increase from 9.16 ± 0.9% for pristine PET, to 35.90 ± 1.6% for PET@G-ODA-7%, which is nearly similar to PET@G-ODA-1% (37.08 ± 2.01%), showing that even a small amount of 1 wt% can interestingly improves the mechanical properties of PET fabrics. It was previously reported that coated GO fabrics show a decrease in Young's modulus compared to uncoated PET fabrics.^27^ The Young's modulus of PET, PET@G-ODA-1%, PET@G-ODA-3%, PET@G-ODA-5% and PET@G-ODA-7%, was calculated from the elastic domain^64^ in each of the stress–strain curves. Young's modulus for the pristine PET is 208.3 ± 0.40 MPa, while it decreases proportionally with increasing the amount of G-ODA loading to 75.2 ± 0.20 MPa for PET@G-ODA-7% (Fig. 14c). This decrease is probably due to the formation of aggregates that leads to discontinuous nanostructure in the PET surface as it was previously seen by SEM.

## 4. Conclusions

The elaboration of PET to PET@G-ODA coated textiles was carried out by dip-coating approach in an organophilic graphene oxide solution. The ODA-functionalized graphene was successfully synthesized by covalently grafting a long chain fatty amine to graphene's surface. The prepared textiles exhibit an enhanced thermal stability and a proportional increase in the contact angle with the amount increasing of G-ODA from 1 to 7%. Compared to the pristine PET, the G-ODA coated PET was found to be highly efficient for oil/water separation. Tensile properties including tensile strength and elongation at break reflected an improvement with G-ODA coating. The PET@G-ODA coated samples show promising results, which make them suitable to be used in different scientific and industrial applications.

## Conflicts of interest

The authors declare that they have no competing interests.

## Supplementary Material
